# Medical Union Number Six

**Published:** 1904-07

**Authors:** 


MEDICAL UNION NUMBER SIX. By WILLIAM HARVEY KING. Sextodecimo, 60 pages. New York: The Monograph Press, 1904. (Price, 35 cents.)

This book is given to the professional world as a satire on unionism and, although the author does not say so in so many words, it doesn't need more than ordinary intelligence to grasp that idea. Without going into any extended criticism of the literary style of the work, out of deference to the feelings of the author, it is rather difficult to guess just why he should have selected so dignified a profession as medicine to illustrate his satire.

The basic idea of the book is impossible. Whatever one's personal opinion may be regarding the benefits or evils of trades' unionism he cannot read of the action of Medical Union No. 6, with its improbable doctors and its impossible "riding delegates," without a feeling of repugnance which later changes to pity for the misguided literary efforts of a promising writer, who so far loses sight of the fundamental principles of medicine as to permit,—even in a satire,—a physician to leave a dying child to choke to death with croup, because croup came under the care of the throat physician, and union rules forbade him,—a chest doctor,—going outside the prescribed limits of his specialty even to save life

That is one of the things that robs the book of its satire and brings it close to the thistle field of assininity. A really good satirical work, to rank among the gems, must have polish and be builded on fact. Medical Union No. 6 lacks both. Yet it is readable, for all that, and it could with much right, occupy a place in a literary museum among the curosities.

